# Congenital insensitivity to pain associated with *PRDM12* mutation: Two case reports and a literature review

**DOI:** 10.3389/fgene.2023.1139161

**Published:** 2023-03-20

**Authors:** Hanrui Yu, Jie Wu, Jinju Cong, Mingxiong Chen, Yifei Huang, Jifeng Yu, Liqiang Wang

**Affiliations:** ^1^ Medical School of Chinese PLA, Beijing, China; ^2^ Department of Ophthalmology, The Third Medical Center, Chinese PLA General Hospital, Beijing, China; ^3^ Department of Ophthalmology, Hainan Hospital of Chinese PLA General Hospital, Sanya, China; ^4^ Aier Eye Hospital, Qianjiang, Hubei Province, China; ^5^ School of Medicine, Nankai University, Tianjin, China; ^6^ Department of Ophthalmology, Beijing Children Hospital, Capital Medical University, National Center for Children’s Health, Beijing, China

**Keywords:** Prdm12, insensitivity to pain, corneal disease, self-mutilation behavior, HSAN, hereditary and sensory autonomic neuropathy

## Abstract

**Background:** *PRDM12* is a newly discovered gene responsible for congenital insensitivity to pain (CIP). Its clinical manifestations are various and not widely known.

**Methods:** The clinical data of two infants diagnosed with CIP associated with *PRDM12* mutation were collected. A literature review was performed, and the clinical characteristics of 20 cases diagnosed with a mutation of *PRDM12* were summarized and analyzed.

**Results:** Two patients had pain insensitivity, tongue and lip defects, and corneal ulcers. The genomic analysis results showed that variants of *PRDM12* were detected in the two families. The case 1 patient carried heterozygous variations of c.682+1G > A and c.502C > T (p.R168C), which were inherited from her father and mother, respectively. We enrolled 22 patients diagnosed with CIP through a literature review together with our cases. There were 16 male (72.7%) and 6 female (27.3%) patients. The age of onset ranged from 6 months to 57 years. The prevalence of clinic manifestation was 14 cases with insensitivity to pain (63.6%), 19 cases with self-mutilation behaviors (86.4%), 11 cases with tongue and lip defects (50%), 5 cases with mid-facial lesions (22.7%), 6 cases with distal phalanx injury (27.3%), 11 cases of recurrent infection (50%), 3 cases (13.6%) with anhidrosis, and 5 cases (22.7%) with global developmental delay. The prevalence of ocular symptoms was 11 cases (50%) with reduced tear secretion, 6 cases (27.3%) with decreased corneal sensitivity, 7 cases (31.8%) with disappeared corneal reflexes, 5.5 cases (25%, 0.5 indicated a single eye) with corneal opacity, 5 cases (22.7%) with corneal ulceration, and 1 case (4.5%) with a corneal scar.

**Conclusion:** The syndrome caused by *PRDM12* mutation is a clinically distinct and diagnosable disease that requires joint multidisciplinary management to control the development of the disease and minimize the occurrence of complications.

## Introduction

Pain is a protective perception response to most harmful stimuli. Insensitivity to pain leads to an unguarded body, vulnerable to damage. Congenital insensitivity to pain (CIP) is a group of rare genetic pain loss disorders defined by its congenital onset. Hereditary sensory and autonomic neuropathy (HSAN) is also a genetic pain loss disorder that tends to develop gradually over time. Occasionally CIP and HSAN can overlap as the difference is not clear (Lischka et al., 2022). HSAN has been classified into types I–VIII according to the main mutation genes: *SPTLC1*, *SPTLC2*, *ALT1*, *WNK1*, *SCN9A*, *NTRK1*, *NGFβ*, *SCN11A*, and *PRDM12* (Rotthier et al., 2012; Schwartzlow and Kazamel, 2019). Types Ⅵ, VII, and VIII of HSAN correspond to types I, II, and III of CIP (Schwartzlow and Kazamel, 2019).

*PRDM12* is a newly identified causative gene for CIP. Members of the PRDM protein family have a PR domain and differing numbers of Zn-finger repeats. PRDM proteins regulate gene expression by either directly altering the chromatin structure through intrinsic methyltransferase activity or indirectly by attracting chromatin remodeling complexes (Di Zazzo et al., 2013). The human *PRDM12* gene is localized on chromosome 9 at 9q33-q34, according to the Entrez Gene [Gene ID: 59335]. It has been proved that *PRDM12* plays an important role in human pain perception (Nahorski et al., 2015; Drissi et al., 2020). The nerve growth factor (NGF)/tyrosine receptor kinase A (TrkA) signaling pathway is required for the survival and specification of nociceptors and plays a major role in pain processing. *PRDM12* regulates the expression of NGF receptor TrkA to guide the development of nociceptive sensory neurons (Desiderio et al., 2019).

Mutations in *PRDM12* are currently believed to cause HSAN-VIII and midface toddler excoriation syndrome (MiTES). HSAN is a rare hereditary neuropathy classified into types I–VIII (Rotthier et al., 2012; Schwartzlow and Kazamel, 2019). The mutation of the *PRDM12* gene leads to the autosomal recessive HSAN-VIII type, also known as CIP3. The clinical symptoms vary, including growth delays, anhidrosis, self-mutilation behaviors, and self-injury-induced oral and corneal ulcers. Some injuries could be cured with treatment, while others could lead to lifelong tissue defects if left untreated. For instance, the patient reported by Gaur et al. (2018) suffered from frequent self-mutilation behaviors due to insensitivity to pain. He was only 1 year old when he had severe corneal scarring, a lip defect, and distal phalangeal injury caused by self-mutilation behaviors. Likewise, Moss et al. (2018) also reported a disease associated with *PRDM12* mutations called MiTES. Unlike CIP3, MiTES is a relatively singular clinical presence with mostly scarring in the midface rather than a widespread insensitivity to pain (Moss et al., 2018). Thus, the two syndromes caused by *PRDM12* easily cause appearance damage to patients, which seriously affects the quality of life.

Because the clinical manifestations caused by *PRDM12* mutation are various, a lack of gene analysis as a routine clinical examination makes it difficult to diagnose this disease. Therefore, syndromes caused by a *PRDM12* mutation are prone to be missed. Without early detection and early diagnosis, early treatment and early prevention are impossible. At the same time, due to the diversity of clinical manifestations of such diseases, including corneal ulcers, oral ulcers and infection, multidisciplinary collaborative management is needed, which increases the difficulty of treatment.

Our study hopes to summarize the clinical symptoms and treatment plans of two patients with *PRDM12* mutations admitted to our hospital and 20 patients with *PRDM12* mutations reported in the literature, analyze the impact of related symptoms on patients, formulate corresponding clinical management measures, and systematically elaborate the comprehensive prevention and treatment plan for patients with a *PRDM12* mutation. It provides new ideas and comprehensive knowledge for the diagnosis and treatment of this disease.

## Materials and methodology

Two patients with *PRDM12* mutations confirmed in Beijing Children’s Hospital affiliated with Capital Medical University were included in this study. All the children’s guardians were informed and signed written informed consent. The terms “PRDM12,” “Hereditary sensory and autonomic neuropathy-VIII,” “Congenital insensitivity to pain 3,” and “Midface toddler excoriation syndrome” were searched in the National Library of Medicine of the United States (PubMed) from the time of establishment to October 2022. Inclusion criteria were the discovery of associated pathogenic genes.

### Genetic testing

Genomic DNA was extracted from the peripheral blood of patients to construct a genomic library, and then the exon and adjacent intron regions (50bp) of all human genes were captured by probe hybridization and enriched. The enriched target gene fragments were sequenced by a next-generation high throughput sequencer (Illumina). NextGene V2.3.4 software was used to compare the sequencing data with the human genome hg19 reference sequence provided by the UCSC database, and the coverage of target regions and sequencing quality were evaluated.

## Results

### Case 1

An 11-month female infant presented with recurrent corneal and oral ulcers that had persisted for 6 months. The infant developed corneal ulcers the size of rice grains in both eyes and oral ulcers successively at the age of 5 months. The local hospital treated her for “traumatic ulcers,” but she showed no significant improvement. The sizes of corneal and oral ulcers increased gradually.

The patient’s family had visited several major hospitals for the corneal ulcers. Traditional treatments such as tobramycin, levofloxacin, calf blood deproteinized extract eye gel, ganciclovir eye gel, and interferon eye drops were applied successively with no obvious improvement. The corneal ulcer gradually got worse, as did her oral ulcers. For the oral ulcer, symptomatic treatments were applied with no obvious improvement. The oral bacterial culture results found *Staphylococcus aureus* and *Streptococcus salivarius*, which were susceptible to benzoxicillin. The systemic use of linezolid and ertapenem to control the recurrent infection received little response.

**FIGURE 1:**
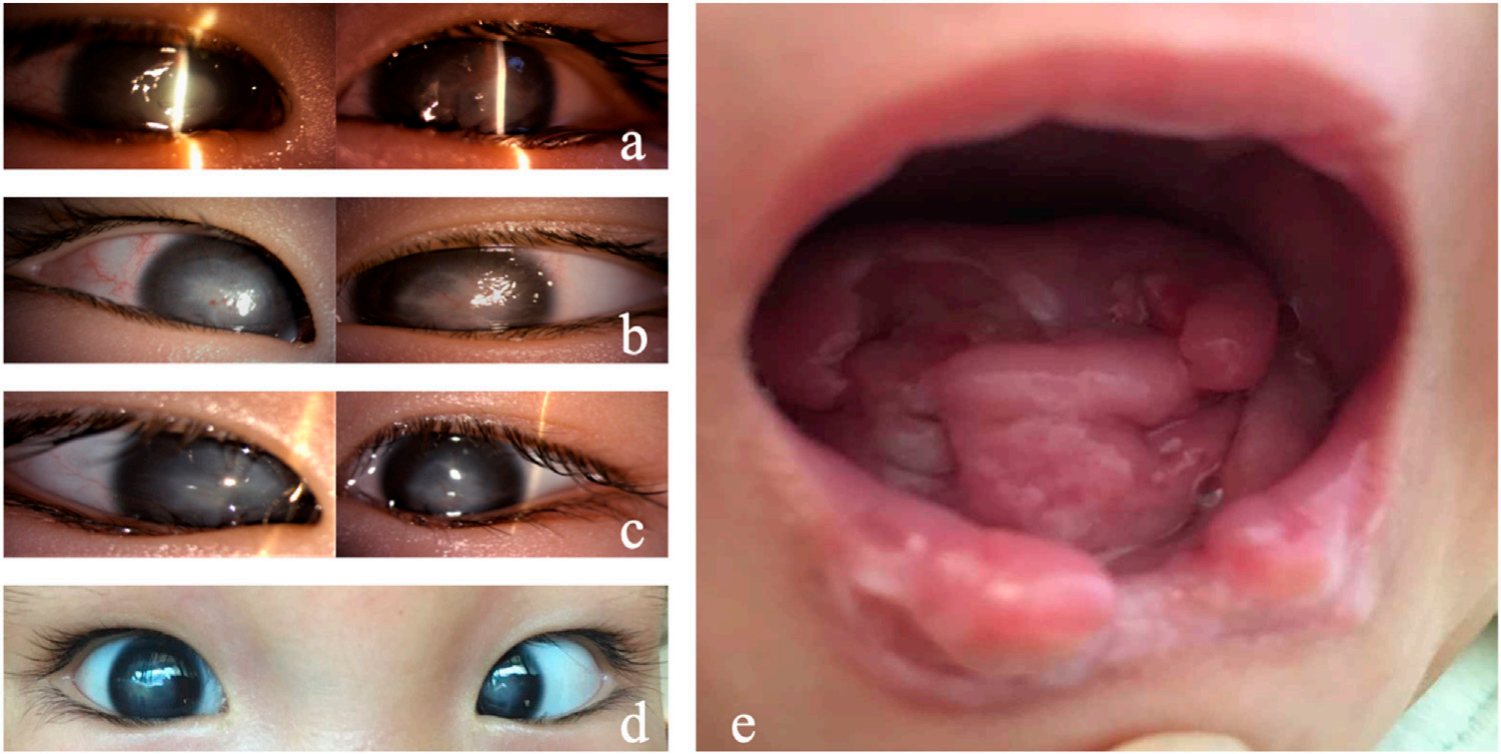
Clinical manifestations of Case 1. **(A)** Both eyes showed conjunctival hyperemia, corneal ulceration, deep stromal infiltration, borderline ambiguity, and opacity. **(B)** On day 7, the corneal ulcers in both eyes gradually healed, and the conjunctival congestion was reduced. **(C)** On day 14, the corneal ulcers healed in both eyes. **(D)** On day 40, conjunctival hyperemia (−) occurred in both eyes, the corneal epithelium was intact, and corneal leukoplakia formed. **(E)** Tongue and lip defects and oral ulcers.

When the patient was admitted to our hospital, the ophthalmic examination found conjunctival congestion and corneal ulcers in both eyes. The corneal ulcers infiltrated the deep stromal layer with an unclear borderline (Figure 1A). The chronic oral ulcer had caused profound defects of tongue and lip (Figure 1E). The medical history showed that the patient had no perspiration after birth, insensitivity to pain, developmental delay, and no obvious joint deformity. Based on the systemic clinical manifestations, we considered that the child had HSAN, so we conducted genetic testing on the child. The report showed a mutation of *PRDM12*. The patient carried pathogenic compound heterozygous variations of c.682+1G > A and c.502C > T (p.Arg168Cys), which were inherited from her father and mother, respectively. The ACMG criteria showed c.682+1G > A is pathogenic, and c.502C > T (p.Arg168Cys) is of uncertain significance. Her parents were not consanguineous in marriage. The diagnosis was confirmed as PRDM12 mutation-related CIP.

As for the treatment, considering the infant with unknown etiology, weak general conditions, fever, and no sweat, surgery is not optimal as the anesthesia is risky. In addition, the penetrating keratoplasty requires intense care after surgery, which could be difficult as she perceives no pain. A strong eye rubbing or a delayed blink could lead to graft damage. After a comprehensive systemic status assessment, we used natamycin eye drops and gatifloxacin eye gel for both eyes and continued anti-infection symptomatic treatment for the whole body. The corneal ulcer improved significantly 2 days later. The oral secretion culture results showed *Candida guilliermondii* infection. After 7 days of ocular antifungal treatment, the corneal ulcer was limited, and conjunctival congestion was relieved. Systemic antifungal treatment with fluconazole injection was added. The patient’s condition gradually stabilized, and the fever was controlled (Figure 1B). After 2 weeks, systemic medication was gradually stopped, and the corneal and oral ulcers gradually healed (Figure 1C). Thereafter, the systemic medical treatment was symptomatic, and the eyes were regularly moisturized with preservative-free, artificial tears (0.3% sodium hyaluronate) eye drops. The cornea remained stable 4 weeks after discharge (Figure 1D).

### Case 2

**FIGURE 2:**
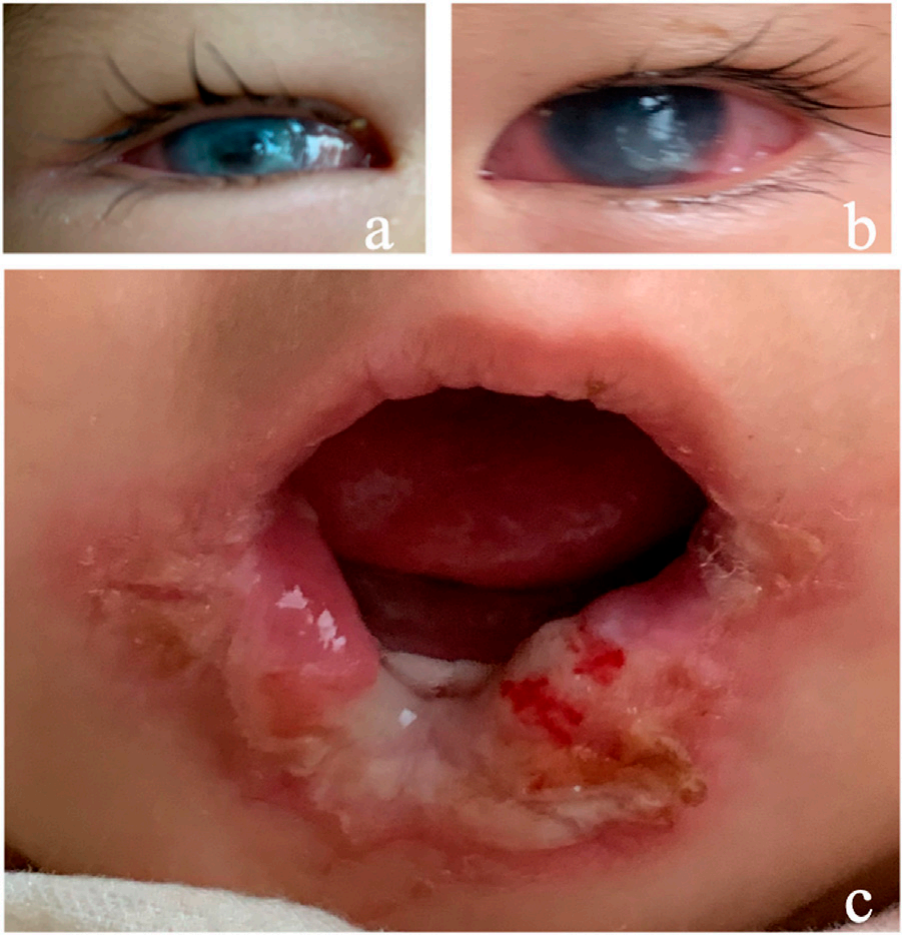
Clinical manifestations of Case 2. **(A,B)** Conjunctival hyperemia, corneal ulcer; **(C)** tongue and lip defects and oral ulcers.

A 15-month female infant presented with corneal opacity in both eyes for 5 months. She came to the hospital with a body temperature of 38.5 °C. The ophthalmic examination showed that the corneal reflex disappeared, tear secretion decreased, conjunctival congestion was present in both eyes, and both eyes showed corneal opacity (Figure 2A,B). Her tongue and lip were defective (Figure 2C). A purulent mossy attachment was noted around the oral cavity, on the oral mucosa and on the tongue surface. She was born at full term. The parents recalled little blinking after her birth. Eight months after birth, the infant manifested continuously sucking and biting the lower lip with no obvious incentives. Large mucosal ulcers were also noticed in the oral cavity. Oral anti-infection treatment and behavioral interventions such as sticking the lower lip with medical tape or usage of a pacifier to correct the habit of biting the lower lip were not effective. At the age of 10 months, her right eye was noticed to be cloudy, and her motor and intellectual development were delayed. Their parents were not consanguineous in marriage. There was no history of genetic family disease. Confirmation of the PRDM12 mutation in the patient was made through telephone follow-up with her family members. The type of mutation is not available, and co-segregation analysis has not been performed in this family. The diagnosis was confirmed as PRDM12 mutation-related CIP.

### Literature review

A total of 11 articles that had reported the *PRDM12* mutation-related disease were retrieved, including 20 cases with detailed case reports. Together with our cases, 22 cases were included. The demographic characteristics and ocular, oral, facial, and skeletal manifestations of the patients were summarized and analyzed. We summarized the clinical manifestations of all patients with *PRDM12* mutations reported so far in Table 1 and summarized the patients with detailed disease descriptions in Table 2. All variations of *PRDM12* are summarized in Table 3.

**TABLE 1:** Review of cases with *PRDM12-*related pain insensitivity.

Authors, years	Number of cases	Sex	Age at presentation	Clinical manifestations	Therapy	Outcome
Chen et al. (2015)	21	Male: 13	30–40 years	Insensitivity to pain and temperature, self-mutilation behavior, mutilation of tongue and lips, mutilation of distal phalanges, loss of corneal reflexes, corneal scarring, recurrent infections, normal intellect	-	-
		Female: 8				
Zhang et al. (2016)	5	Male: 4	23–57 years	Insensitivity to pain, self-mutilation behavior, mutilation of tongue and lips, mutilation of distal phalanges, loss of corneal reflexes, decreased tear secretion, corneal abrasions, recurrent infections, normal intellect	-	Two individuals had no useful vision in one eye, and in one, multiple corneal grafts had failed
		Female: 1				
Saini et al. (2017)	1	Male	2 years	Insensitivity to pain and temperature, self-mutilation behavior, mutilation of tongue and lips, reduced blink, global development delay, normal sweating, and tear overflow	-	-
Elhennawy et al. (2017)	1	Male	8 months	Insensitivity to pain and temperature, self-mutilation behavior, loss of corneal reflexes, recurrent infections, reduced sweat, and tear production. Oral manifestations: premature loss of teeth associated with dental traumata and self-mutilation, severe soft tissue injuries, dental caries and submucosal abscesses, hypomineralization of primary teeth, and mandibular osteomyelitis	Anti-infection treatment	-
Moss et al. (2018)	5	Male: 4 Female: 1	1–4 years	Self-mutilation behavior, global developmental delay, skin excoriations on nose, forehead, medial aspect of eyes, and chin (MiTES)	-	-
Gaur et al. (2018)	1	Male	1 year	Insensitivity to pain, self-mutilation behavior, mutilation of tongue and lips, mutilation of distal phalanges, loss of corneal sensation, corneal scarring, normal intellect	Corneal transplant	-
Navya et al. (2019)	1	Male	1 year	Insensitivity to pain, self-mutilation behavior, mutilation of tongue and lips, mutilation of distal phalanges, recurrent infections, global development delay	Systemic anti-infection treatment, limit biting	Healing of lesions in the thumb and oral mucosa
Kaur et al. (2020)	1	Male	1 year	Insensitivity to pain and temperature, self-mutilation behavior, mutilation of tongue and lips, corneal opacification, decreased lacrimation	Permanent left blepharoplasty, right corneal transplantation	-
Suthar et al. (2022)	2	Male: 1	6 months	Insensitivity to pain and temperature, self-mutilation behavior, persistent rhinitis, mutilation of tongue and lips, corneal ulcer, global development delay	-	-
		Female: 1				
Kusumesh et al. (2022)	1	Female	2 years	Insensitivity to pain and temperature, self-mutilation behavior, mutilation of distal phalanges, corneal opacification, loss of sweating and tears, abnormal tooth development	-	-
Elsana et al. (2022)	3	Male: 2	3–11 years	reduced tear production, loss of corneal reflexes, disappearance of corneal blink reflex, corneal opacification, corneal ulcer, corneal scarring	Lateral tarsorrhaphy, corneal transplant	-
		Female: 1				
Index children	2	Female: 2	1–2 years	Insensitivity to pain, self-mutilation behavior, mutilation of tongue and lips, loss of corneal reflexes, corneal opacification, corneal ulcer, decreased lacrimation, recurrent infections, global developmental delay	Systemic anti-infection treatment	Case 1 can now live normally without affecting vision

**TABLE 2:** Clinical features of patients diagnosed with *PRDM12* mutations.

Authors, years	Number of cases	Sex	Age at presentation	Clinical manifestation													
				Insensitivity to pain	Self-mutilation behavior	Mutilation of tongue and lips	Mid-facial lesion	Mutilation of distal phalanges	No sweating	Recurrent infections	Ocular manifestations						Global developmental delay
											Reduced tear production	Global developmental delay	Disappeared corneal reflexes	Corneal opacity	Corneal ulcer	Corneal scarring	
Zhang et al. (2016)	5	Male: 4	23–57 years	5	5	3	-	3	1	5	5	-	5	-	-	-	-
		Female: 1															
Saini et al. (2017)	1	Male	2 years	1	1	1	-	-	-	-	-	1	-	-	-	-	1
Elhennawy et al. (2017)	1	Male	8 months	1	1	1	-	-	1	1	-	-	1	-	-	-	-
Moss et al. (2018)	4	Male: 4	1–4 years	-	4	-	4	-	-	-	-	-	-	-	-	-	-
Gaur et al. (2018)	1	Male	1 year	1	1	1	-	1	-	-	-	1	-	-	-	1	-
Navya et al. (2019)	1	Male	1 year	1	1	1	-	1	-	1	-	-	-	-	-	-	-
Kaur et al. (2020)	1	Male	1 year	1	1	1	-	-	-	1	1	1		1	-	-	-
Suthar et al. (2022)	2	Male: 1	6 months	1	1	1	-	-	-	-	-	-	-	-	1	-	2
		Female: 1															
Kusumesh et al. (2022)	1	Female	2 years	1	1	-	1	1	-	1	1	-	1	1	-	-	-
Elsana et al. (2022)	3	Male: 2	3–11 years	-	-	-	-	-	-	-	3	2	-	2.5	3	-	-
		Female: 1															
Index children	2	Female: 2	1–2 years	2	2	2	-	-	2	2	1	1	-	1	1	-	2

**TABLE 3:** Mutation sites identified in a patient with a *PRDM12* gene mutation.

Authors, years	Number of cases	Sex	Age at presentation	Mutation sites in the *PRDM12* gene
Zhang et al. (2016)	5	Male:4 Female: 1	23–57 years	-
Saini et al. (2017)	1	Male	2 years	homozygous mutation c.224–2A > G in the *PRDM12* gene
Elhennawy et al. (2017)	1	Male	8 months	homozygous mutation c.516G>C (p. Glu172Asp) in the *PRDM12* gene
Moss et al. (2018)	4	Male: 4	1–4 years	heterozygous for a partial gene deletion of *PRDM12* involving at least exon 5; heterozygous expansion of the *PRDM12* polyalanine tract to 18/17 alanine residues
Gaur et al. (2018)	1	Male	1 year	homozygous mutation
Navya et al. (2019)	1	Male	1 year	mutation in exon 5 of *PRDM12* gene
Kaur et al. (2020)	1	Male	1 year	a heterozygous, missense variation in exon 2 of *PRDM12* gene (chr9:g.133542114G>C) that results in the amino acid substitution of arginine for glycine at codon 115 (p.Gly115Arg) and another heterozygous, missense variation in exon 3 of *PRDM12* gene (chr9:g.133543652C>A; Depth: 63) that results in the amino acid substitution of lysine for asparagine at codon 174 (p.Asn174Lys)
Suthar et al. (2022)	2	Male: 1 Female: 1	6 months	Female: a homozygous missense variant (c.451G>A; p.Asp151Asn)
Kusumesh et al. (2022)	1	Female	2 years	homozygous mutation in the *PRDM12* gene on chromosome 9q34
Elsana et al. (2022)	3	Male: 2 Female: 1	3–11 years	homozygous for the *PRDM12* variant: Chr9:133543585 (GRCh37/hg19); c.455C>A (p.Ala152Asp)
Index children	2	Female: 2	1–2 years	heterozygous variations of c.682+1G > A and c.502C > T (p.Arg168Cys)

Among the 22 patients, there were 16 males (72.7%) and 6 females (27.3%). The age of onset ranged from 6 months to 57 years. The prevalence of clinic manifestation was 14 cases with insensitivity to pain (63.6%), 19 cases with self-mutilation behaviors (86.4%), 11 cases with tongue and lip defects (50%), 5 cases with a mid-facial lesion (22.7%), 6 cases with distal phalanx injury (27.3%), 11 cases of recurrent infection (50%), 3 cases (13.6%) with anhidrosis, and 5 cases (22.7%) with global developmental delay. The prevalence of ocular symptoms was 11 cases (50%) with reduced tear secretion, 6 cases (27.3%) with decreased corneal sensitivity, 7 cases (31.8%) with disappeared corneal reflexes, 5.5 cases (25%, 0.5 indicated a single eye) with corneal opacity, 5 cases (22.7%) with corneal ulceration, and 1 case (4.5%) with a corneal scar.

## Discussion

CIP3 is a rare inherited pain loss disorder with various clinical manifestations. As the symptoms varied, including insensitivity to pain, self-mutilation behaviors, recurrent infections, and self-injury-induced oral and corneal ulcers, doctors tend to only notice a single symptom and overlook the overall presentation, resulting in misdiagnosis and missed diagnosis.

Genetic analysis, as the only means of genetic disorder diagnosis, can accurately locate the mutated gene, identify the type of disease, and give clues in the prenatal examination. Studies have shown that the number of newborns diagnosed with HSAN-III has decreased significantly over the past decade with the help of prenatal testing (Couzin-Frankel, 2010). Fetuses with *PRDM12* mutations may also be identified prenatally by such means. A comprehensive understanding of the *PRDM12* mutation-related disease could be helpful for early diagnosis and treatment (Imhof et al., 2020). No association was found among the *PRDM12* mutation sites in the 22 reported patients. However, the mutation may affect the structure of the protein and affect the distribution of pain perception. The aim of this study is to systematically review *PRDM12* mutation-related CIP based on our two patients and the 20 cases reported in the literature. The overall findings of our study demonstrated that CIP due to *PRDM12* mutations usually resulted in pain insensitivity, facial and limb defects, and recurrent infections, which significantly damaged children’s growth.

### Insensitivity to pain and other pain-related features

Pain insensitivity is one of the most distinctive features of all diseases caused by *PRDM12* mutations. The insensitivity to pain caused by *PRDM12* mutation leads to the defect of the nociceptors during embryonic development (Chen et al., 2015; Landy et al., 2021; Rienzo et al., 2021). With the inability to feel pain, patients often unconsciously show some self-mutilation behaviors. Self-inflicted injuries are more vulnerable to infection and decreased immunity. Recurring infections are the outcome. This causes great difficulties for parents in looking after their children. Self-mutilation behaviors cause defects in various body parts at an early age, which can have lifelong effects on life and appearance. Treatment was limited to plastic surgery, such as functional alginate dressings (Jones et al., 2006), autologous skin grafts (Hu et al., 2015), and cell-based wound healing therapy (Rodrigues et al., 2019). Another difficulty in treating CIP3 is recurrent infection. Because the patient cannot perceive the injury, the healing process may be accompanied by new wounds.

### Ophthalmological complications

Patients with *PRDM12* mutations present with reduced tear secretion, corneal abrasions, and loss of corneal reflexes, resulting in keratitis and corneal scarring (Chen et al., 2015; Zhang et al., 2016). We found that almost every patient had some degree of corneal injury, indicating that the corneal symptoms deserve attention.

There is no clear report in the literature on why patients with *PRDM12* mutations have different degrees of eye damage, and we expect to explore this next. Because HSAN autosomal recessive patients often develop the disease at an early age, before irreversible damage is caused, eye symptoms tend to give us a better warning (Schwartzlow and Kazamel, 2019). For example, patients with dry eye signs on the ocular surface, corneal opacity or decreased corneal sensitivity, if further aggravated, such as corneal opacity or increased secretions, should seek medical attention in time to minimize the damage.

As shown in Table 1, when selecting the treatment plan, except for the two patients reported by us, conservative treatment was adopted, and most of the patients with detailed reports of their disease were controlled through corneal transplantation and other operations. In Case 1, penetrating keratoplasty was considered to repair corneal ulcers during a visit to another hospital. Yagev et al. (1999) reported that a child with binocular corneal ulcers caused by painless syndrome was treated with penetrating keratoplasty under good overall condition, but the postoperative effect was poor. Corneal transplantation is a relatively complicated ophthalmic operation that imposes high requirements on patients’ general condition and postoperative nursing (Tan et al., 2012). CIP patients tend to have poor systemic status due to recurrent infections. Moreover, uncontrolled eye rubbing and eye damage will inevitably occur after surgery, resulting in artificial transplant failure. Therefore, after carefully considering the circumstances and full communication with the parents, we adopted conservative treatment. The patient’s vision recovered well, and her daily life was not affected. For Case 1, controlling fever and infection in the early stage is the focus of treatment. When the physician could not find the cause, we started from the local symptoms of the eye and adopted antifungal treatment, which not only avoided the corneal perforation but also effectively controlled the systemic condition of the child.

## Others

CIP3 can also lead to global developmental delays. Although the proportion is relatively low, it has a great impact on the growth of children. If the doctor suspects this disease when treating the patients, parents should be reminded to assess the intelligence of their child and avoid missed diagnoses.

MiTES, meanwhile, could be an early warning sign of *PRDM12*-CIP. MiTES patients often present with pathological itching in the mid-face, an inability to manage the damaged area due to pain insensitivity, and mid-facial lesions after persistent scratching (Moss et al., 2018). Although there is no other evidence of damage to *PRDM12*-CIP in the disease profile of MiTES patients, four of five had mutations in *PRDM12*. MiTES, therefore, should be considered if a child is observed unconsciously scratching the midface area. Medical attention is needed to avoid the possibility of MiTES’ progression to *PRDM12*-CIP. Facial defects often cause the appearance of patients with terror, and their normal growth and integration into society cause great difficulties. Attention should also be paid to the mental health status of this group (McCarron et al., 2021).

## Conclusion

Patients with a *PRDM12* gene mutation benefit from early detection and diagnosis. Early intervention can greatly control the progression of the disease so that the appearance and vision of the patients will not be affected to the greatest extent. Secondly, different treatment measures should be implemented according to age stages and the severity of the disease, and symptomatic treatment against infection should always be maintained. Finally, the patient’s family should attend to the patient’s psychological problems.

## Data Availability

The original contributions presented in the study are included in the article/Supplementary material; further inquiries can be directed to the corresponding authors.
